# Clinical Drug-Resistant Cutaneous Tuberculosis Presenting as Lupus Vulgaris Ulcerative Type in an Indian Female With Poncet’s Disease: A First-of-Its-Type Report

**DOI:** 10.7759/cureus.57358

**Published:** 2024-03-31

**Authors:** Sankalp Yadav

**Affiliations:** 1 Medicine, Shri Madan Lal Khurana Chest Clinic, New Delhi, IND

**Keywords:** diascopy, biopsy, clinical drug resistant tuberculosis, mtb (mycobacterium tuberculosis), poncet’s disease

## Abstract

Tuberculosis of the skin is rare and a difficult diagnosis. Moreover, recurrent episodes of mycobacterial infection in the skin with Poncet’s disease are rarely reported in females. Herein, the first of its type case of clinical drug-resistant tuberculosis of the skin in an Indian female is presented. She had a history of cutaneous tuberculosis five times in the past. At the sixth time, she came with complaints of an ulcerative lesion over her right forearm and cubital fossa with left knee swelling. The paucibacillary nature of the infection made the diagnosis exceedingly challenging. However, a detailed clinical examination with a suspicion of drug resistance resulted in management with significant clinical improvement.

## Introduction

India bears a significant burden of tuberculosis, with the largest number of patients globally [1]. Since the 1980s, there has been a rise in tuberculosis incidence, including cutaneous tuberculosis, attributed to factors such as HIV, multidrug resistance, increased use of immunosuppressive drugs, rising migration rates, and a decline in tuberculosis control efforts [2]. Extrapulmonary tuberculosis comprises 20% of all tuberculosis cases, with cutaneous tuberculosis representing 1.5% of extrapulmonary tuberculosis cases and about 0.9% of dermatology outpatient attendees [3].

Cutaneous tuberculosis is an uncommon form of tuberculosis, with 5.9 incidents reported for every 1,000 people. The overall prevalence of cutaneous tuberculosis is 0.25-0.6%, based on multiple Indian studies [4]. It may be brought on by *Mycobacterium bovis*, the *Mycobacterium tuberculosis *complex, or, in rare cases, the Bacillus Calmette-Guérin vaccine [5]. It can show up clinically in a variety of ways, depending on the immune system of the individual, the environment, and the type of exposure [4]. In India, the most frequent form of skin tuberculosis in adults is lupus vulgaris (75%), but in children, scrofuloderma is more common. People with either a moderate or high level of immunity can be affected by lupus vulgaris, a paucibacillary, chronic, progressive, post-primary type of cutaneous tuberculosis [4].

Herein, an exceedingly rare case of a young Indian female who had a thick, scaly lesion over her right arm and forearm is presented. The case is unique as there was no pulmonary involvement or history of contact with tuberculosis, but she had five episodes of the same condition in the last 17 years, which made the diagnosis challenging in an endemic country. Moreover, a diagnosis of clinical drug-resistant cutaneous tuberculosis was made and management was planned.

## Case presentation

A 26-year-old non-diabetic, married Indian female from a low socioeconomic background reported to the outpatient department in October 2022 as a referral case. She had complaints of two non-healing, gradually progressing ulcerative lesions over the right forearm and cubital fossa for one month, with occasional itching and scanty, foul-smelling pale-yellowish discharge. She had no history of trauma, skin diseases, immunocompromised illness, immunosuppressive drugs, or any other constitutional signs and symptoms of tuberculosis. She was a housewife living separately from her husband. Moreover, there was a history of tuberculosis in the family (her brother received antituberculous treatment for pulmonary tuberculosis). Her personal history was unremarkable.

Her past history was remarkable for multiple episodes of cutaneous tuberculosis, as detailed in Table 1.

**Table 1:** Past treatment history of the patient

Year	Diagnosis	Outcome	Duration of treatment
2007	Lupus vulgaris	Treatment complete	168 days
2014	Lupus vulgaris	Treatment complete	168 days
2017	Lupus vulgaris	Treatment complete	168 days
2020	Lupus vulgaris	Treatment complete	168 days
2022 (January-June)	Lupus vulgaris	Treatment complete	168 days

During her last five episodes, there was a marked reduction in the size of the lesion when she was on antituberculous drugs (rifampicin, isoniazid, ethambutol, and pyrazinamide). However, the lesions continued to grow once the treatment was completed, only to be diagnosed again as lupus vulgaris, involving the right arm and forearm and progressing towards the hand. Further, there was a fixed-flexion deformity at the right elbow joint, which happened due to contractures of the skin in the past episodes.

**Figure 1:**
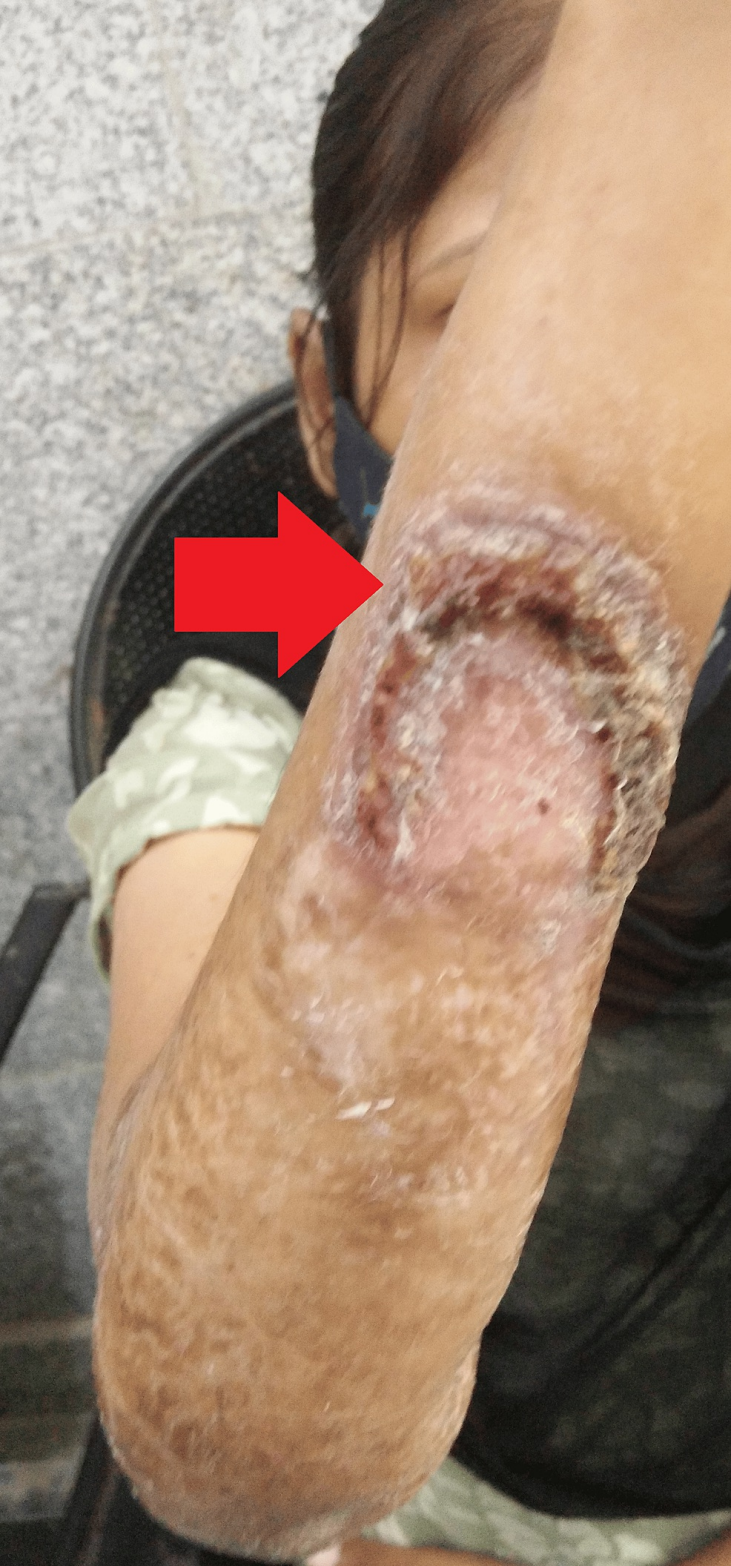
Gross image of the right upper limb showing the lesion

**Figure 2:**
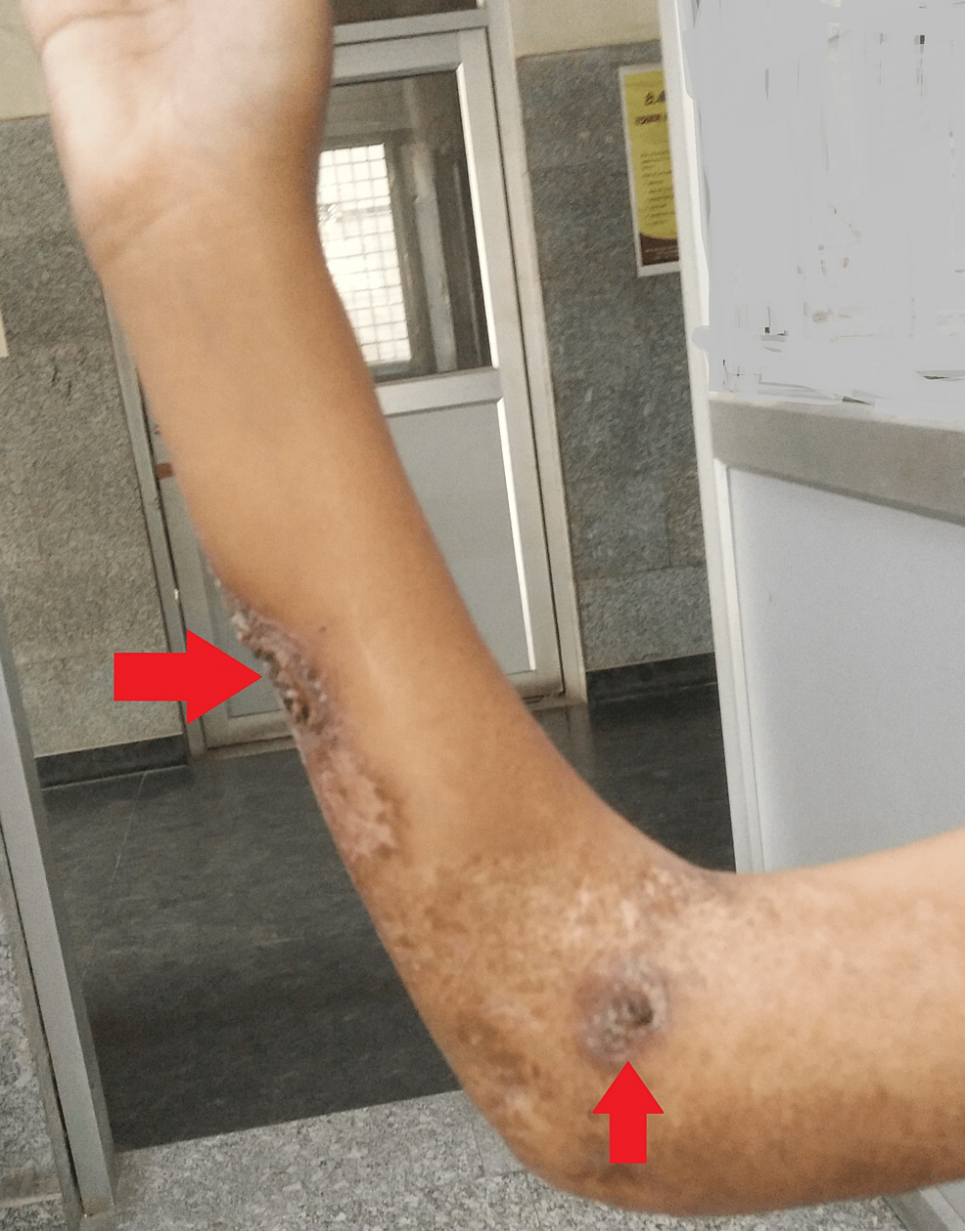
Gross image of the right upper limb showing the lesions

Local examination revealed a large ulcerative lesion with a central clearing of about 8 cm x 7 cm over the ventral surface of the right forearm. Another 1 cm x 1 cm lesion was seen in the right cubital fossa (Figures 1-2).

**Figure 3:**
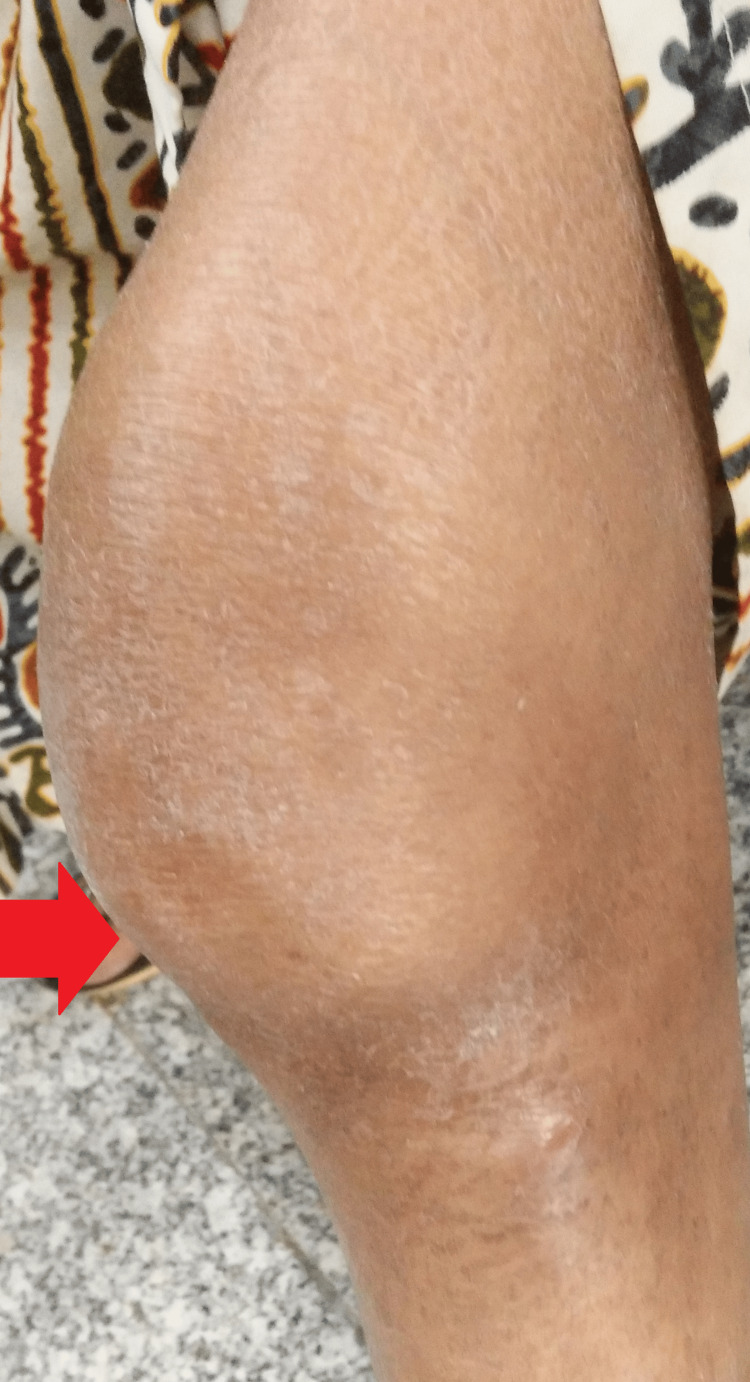
Swollen left knee joint

Further, the local examination of the left knee was suggestive of a swollen, tender joint with a painful and restricted range of movements without any remarkable skin lesions (Figure 3).

**Figure 4:**
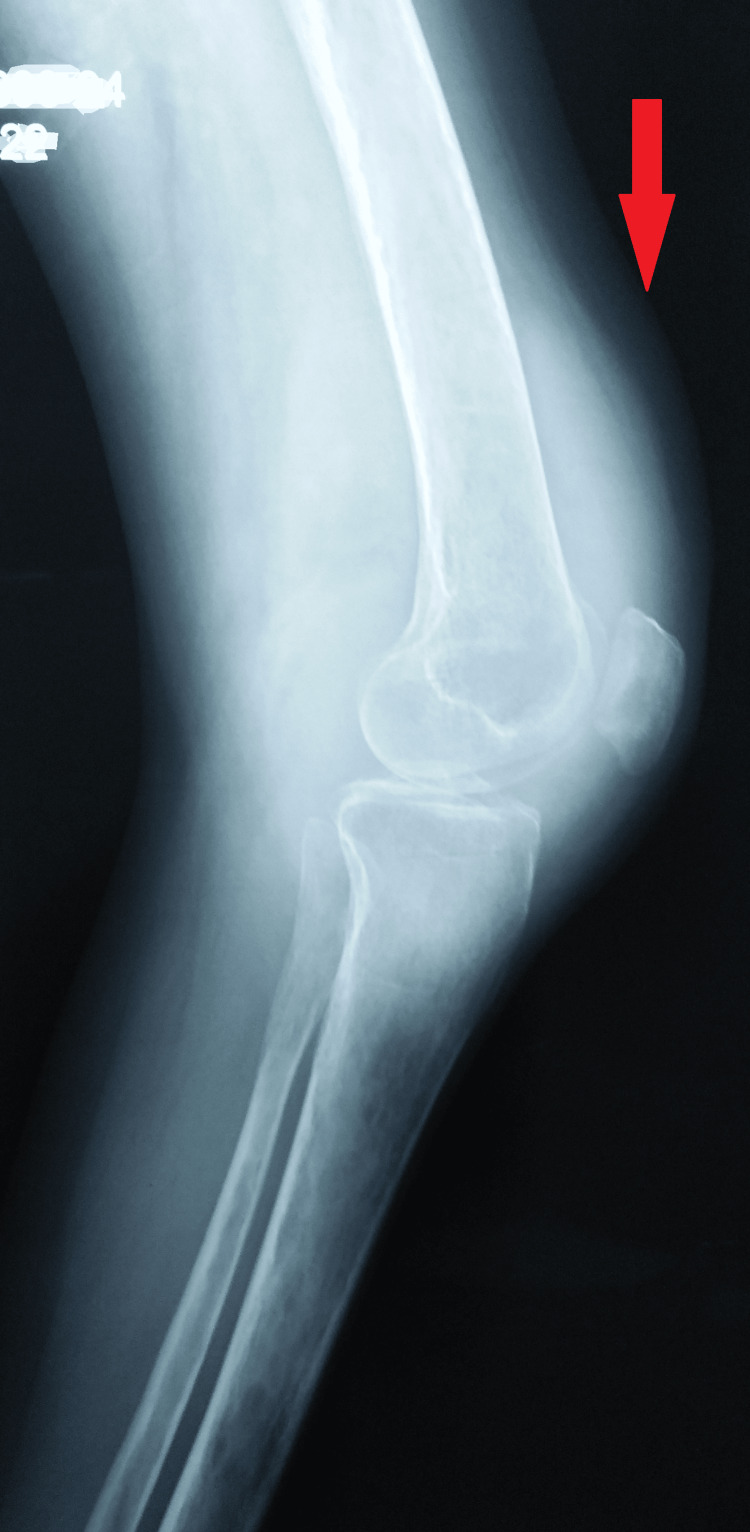
A plain radiograph of the swollen knee not suggestive of any erosion

She was thin, pale, and ectomorphic, but hemodynamically stable, with a weight of 29 kg. She did not have hepatosplenomegaly, genitourinary abnormalities, or abnormal lymph node enlargement. Tests for liver function, HIV, rheumatoid arthritis factor, antinuclear antibody test, electrolytes, and total blood count all came back normal. Her erythrocyte sedimentation rate (49 mm/h) was marginally higher, and her hemoglobin was 7.6 g/dL. The Mantoux test was 30 x 30 mm of induration. A radiograph of the knee was not suggestive of any erosion, but mild suprapatellar bursal thickening was seen in the left knee on ultrasonography; however, a chest radiograph revealed normal results. A synovial fluid biopsy of the left knee joint was unremarkable (Figure 4).

Diascopy showed an apple jelly color at the periphery. A biopsy of the skin lesions revealed mild hyperkeratosis, focal parakeratosis, and mild acanthosis. Dermis showed mild interstitial, perivascular, and periadnaxal inflammatory infiltrates mainly composed of lymphocytes, histiocytes, and multinucleate giant cells. Many epitheloid cell granulomas were noted as suggestive of cutaneous tuberculosis, i.e., lupus vulgaris. However, Ziehl-Neelsen staining for acid-fast bacilli was negative. Besides, the culture of the specimen and polymerase chain reactions for *Mycobacterium tuberculosis *were negative.

A team of experts at the tertiary nodal care center advised management per the national guidelines for clinical drug-resistant tuberculosis. This was based on the facts, as there were five past episodes with temporary relief when the antituberculous treatment was given, and the lesions relapsed after the treatment stopped. Also, the cutaneous lesions are paucibacillary in nature; therefore, detection of *Mycobacterium tuberculosis* and any resistance against the first or second line antituberculous drugs is not always feasible. After an unremarkable pre-treatment evaluation, she was initiated on an all-oral longer regimen per the national guidelines (Table 2).

**Table 2:** An all-oral longer regimen as per her weight

Drug	Dose	Route of administration	Duration	Frequency
Bedaquiline	400 mg	Per oral	2 weeks	Daily
	200 mg	Per oral	22 weeks	Alternate day
Linezolid	300 mg	Per oral	18 months	Daily
Clofazimine	100 mg	Per oral	18 months	Daily
Moxifloxacin (high dose)	400 mg	Per oral	18 months	Daily
Cycloserine	500 mg	Per oral	18 months	Daily
Pyridoxine	100 mg	Per oral	18 months	Daily

**Figure 5:**
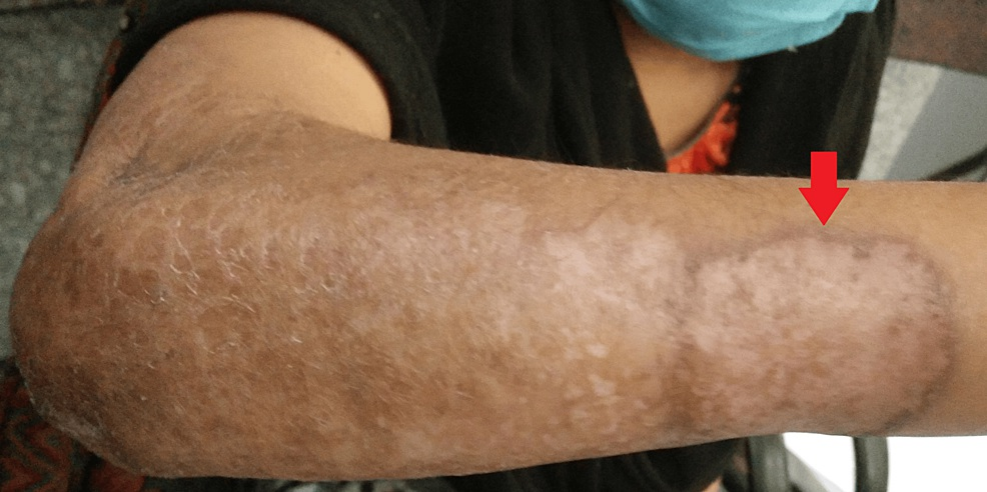
Gross image showing complete resolution of the lesion

**Figure 6:**
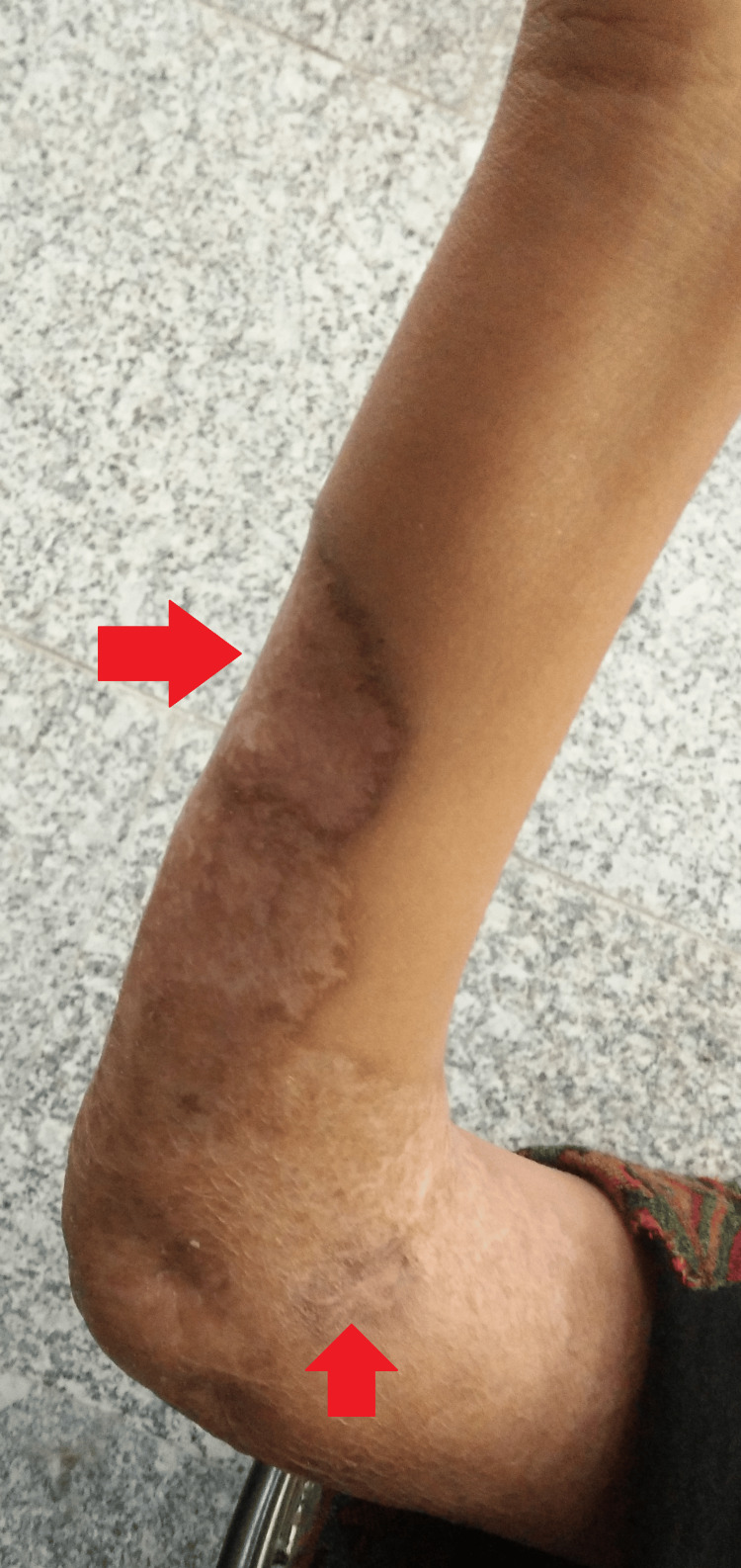
Gross image showing complete resolution of lesions

As she had anemia from a chronic disease, iron supplementation was added, and the dose of linezolid was kept at 300 mg throughout her course. Additionally, she was counseled for timely follow-ups and a hygienic lifestyle. The results were remarkable, with healing of the right arm lesions and complete resolution of left knee joint swelling (at two months). This confirmed the diagnosis of Poncet’s disease of the left knee joint (Figures 5-6).

## Discussion

The diagnosis of cutaneous tuberculosis could be complex [6,7]. Its foundation is made up of either absolute or relative criteria [7]. A positive polymerase chain reaction, tissue culture, or guinea pig inoculation result is the only way to positively identify *Mycobacterium tuberculosis* [6,7]. But it's a difficult task since cutaneous tuberculosis has paucibacillary features [4]. Additional relative criteria that are available include a thorough history and evaluation of lesions, the presence of acid-fast bacillus on lesions, the detection of active tuberculosis in other organs, the finding of a tuberculous granuloma on histopathological examination, a positive tuberculin test, and responsiveness to antituberculous drugs [7].

Lupus vulgaris, named for its ulcerating nature, is a rare, chronic form of tuberculosis. It typically spreads internally through the bloodstream or lymphatic system, occasionally through external sources like infected droplets [4]. Those sensitized to *Mycobacterium tuberculosis *often develop this form of cutaneous tuberculosis [7]. Lupus vulgaris can manifest in various clinical forms, including hypertrophic, plaque-like, tumor-like, papular or nodular, and ulcerative. Less common forms include atrophic and mutilating types [8].

This condition commonly affects the face and neck, particularly in young individuals. However, when lesions occur elsewhere on the body, diagnosis and management become more complex [9]. While facial involvement is common in Western countries, Indian populations typically experience lesions on the buttocks and extremities, as seen in the present case [4].

The hallmark of lupus vulgaris is an unnoticed, scaly plaque known as a lupoma, which develops when reddish-brown, soft-textured papules fuse together [4]. As the plaque gets worse, new papules appear peripherally [9]. Blanching the lesions under diascopic pressure gives them a pale brownish-yellow color, akin to "apple jelly" [4]. Furthermore, distinctive tubercles, either with or without caseation, will usually be revealed by a histological examination [9]. However, polymerase chain reaction testing, commonly used for diagnosis, has limited sensitivity and specificity in lupus vulgaris due to its low bacillary burden. The insertion sequence IS6110, a target for polymerase chain reaction, exhibits variable sensitivity (70-90%) and specificity (90-95%) in research laboratories, with additional dot-blot processes enhancing its accuracy. Culture results are typically negative, with only a 6% positivity rate observed in lupus vulgaris cases [4,9].

Poncet’s disease, also known as tuberculous rheumatism, is a form of reactive arthritis linked to tuberculosis occurring elsewhere in the body. First described by Antonin Poncet in 1897, it is believed to result from an immune cell-mediated reaction to tuberculoprotein, leading to inflammation in the joints. Sharma and Pinto in 2015, based on features observed in 23 patients, gave diagnostic criteria as essential, major, and minor for the diagnosis of Poncet’s disease [10]. The present case had two essential major and two minor criteria.

Management is essentially medical, with antituberculous drugs in histopathologically confirmed cases. In cases where the diagnosis is challenging, a treatment trial of triple antituberculous therapy may be taken into consideration. Usually, the prognosis is good, and responses are seen earlier (4-6 weeks) as compared to other extrapulmonary tuberculosis [4]. However, untreated cases could end up with life-threatening outcomes [11].

According to Ramesh et al., when there is a strong clinical suspicion of cutaneous tuberculosis in a patient and other potential causes have been investigated with less likelihood and the patient does not respond to first-line antituberculous drugs, a trial of second-line drugs may be considered. Although this approach is empirical and may be subject to misuse, in settings where facilities allow, culture and drug sensitivity tests should be conducted before initiating second-line antituberculous therapy. However, even if culture results are negative, it's advisable to continue the drugs to prevent disease worsening. If there is no response within a reasonable period, typically two months, the diagnosis should be reassessed. The present case has shown remarkable improvement at two months; however, she is still on treatment, and the results will be assessed during follow-ups for any evidence of recurrence [12].

With a reported incidence of 0.5-10.5%, malignant tumors are known to exist in lupus vulgaris, with squamous cell carcinoma being the most prevalent kind [4]. In addition, contractures, tissue damage, and disfigurement are consequences of lupus vulgaris lesions, which were present in this case [11].

A similar case was presented by Olson et al. (2007), where the patient had three episodes of cutaneous tuberculosis only to be diagnosed with extensively drug-resistant tuberculosis the fourth time [13]. The present case had five episodes in the past, and only at the sixth time was she diagnosed with clinical drug-resistant tuberculosis, thereby making it the first of its type. However, unlike theirs, this case has taken antituberculous treatment under the directly observed treatment strategy of the National Tuberculosis Elimination Program in her last five episodes [14].

## Conclusions

To conclude, a case like the one presented here requires a strong suspicion of *Mycobacterium tuberculosis *being drug-resistant. The paucibacillary nature of cutaneous tuberculosis stresses the need for policies when the bacteria are not isolated from the specimens and exact drug resistance is not available upfront. Also, it is essential that similar cases be reported so as to create awareness about managing these diagnostic challenges to prevent unfavorable treatment outcomes.
